# Food systems in protracted crises: examining indigenous food sovereignty amid de‐development in Kashmir

**DOI:** 10.1111/disa.12666

**Published:** 2024-10-30

**Authors:** Mehroosh Tak, Sardar Babur Hussain, Haris Zargar, Lauren J. Blake

**Affiliations:** ^1^ The Royal Veterinary College United Kingdom; ^2^ Independent Researcher; ^3^ International Institute of Social Studies of Erasmus University Rotterdam the Netherlands; ^4^ University of Bristol United Kingdom

**Keywords:** agrarian change, indigenous food systems, militarisation, protracted crises, settler‐colonialism

## Abstract

How do protracted crises shape indigenous food systems, and what are their ramifications for food and nutritional security? Building on decolonial and interdisciplinary research approaches, this paper assesses the consequences of militarised violence for Kashmir's food system. We document the impact of settler‐colonialism and conflict‐induced agrarian changes on delocalisation of diets. The protracted nature of the crises has two key implications for changes in dietary patterns. First, land control over common land dispossesses the local population and hinders food production. Second, disenfranchisement from (agricultural) land has led to increased reliance on markets that are flooded by imported foods as local production declines. The paper argues that the state plays an important role in food system changes by destroying local patterns of food production and consumption. Slow violence and agrarian de‐development serve as tools to de‐develop the local food system. Indigenous food cultures form part of everyday resistance and resilience that are operationalised as mitigation and adaptation strategies to address food insecurity.

## 1 INTRODUCTION

Indigenous food systems can be defined as ‘systems of cultivation, processing, storage, trade, and consumption, which are specific to particular geographic regions, and whose origins generally pre‐date large‐scale industrial agriculture’ (Saxena et al., 2016). These systems are fundamental to ensure food security and sovereignty, nutrition, and health for communities facing prolonged, multidimensional pressures of political conflict and climate emergencies. This study assesses the impact of militarised violence on the indigenous food system in Indian‐administered Kashmir. In this contentious Himalayan region, the predominant state‐driven narrative is that local food production is inadequate to feed the population, and the shortfall should be imported. Despite the well‐studied phenomenon of militarised violence in Kashmir, there is little research that explicitly links the matter of protracted crises^1^ to food systems, in Kashmir and other parts of the world. This raises important questions about the processes of agrarian change and their relationship with protracted crises. It is thus pertinent to ask whether existing frameworks are adequate to capture the conflict‐related changes affecting indigenous food systems. We also assess: how agrarian change processes are intimately related to the dynamics of settler‐colonialism or vice versa; how protracted crises shape indigenous food systems; and what the repercussions are for the food and nutritional security of the local communities. We argue that a state‐led de‐development strategy, especially in the form of slow violence and agrarian change, is a key driver of food system change that fuels delocalisation of Kashmiri diets. The paper fills existing blind spots in mainstream food systems and agrarian change literature that tend to ignore the ramifications of settler‐colonialism and agrarian transformation for food systems.

The rest of the paper is structured as follows. The next section provides a critique of existing literature on indigenous food systems in protracted crises. The following two sections describe the study site and the research methods, respectively. The analytical section starts by characterising the local food system in Kashmir, first by historicising land reform since the 1950s and second by analysing how it has influenced the current food system. It goes on to unpack the impact of militarisation and the subsequent consequence for access to land that has crucial implications for Kashmiri diets. Then, we document the use of slow violence on the Kashmiri food system by investigating the impact of land dispossession. The final analysis section maps everyday forms of resistance by focusing on the inherent resilience of the Kashmiri food system.

## 2 LIMITATIONS OF EXISTING ANALYTICAL FRAMEWORKS FOR FOOD SYSTEMS IN PROTRACTED CRISES

Existing scholarship^2^ on food systems ignores the role of settler‐colonialism in de‐developing indigenous food systems and broadly focuses on food security rather than the whole system. This section analyses the blind spots in existing literature by setting the connections between violent state formations and processes of capitalist accumulation and the limitations of primitive accumulation as a paradigm to assess food systems in protracted crises.

The mainstream literature on conflict and agrarian questions states that the solution to preventing and overcoming conflict is economic growth and development (Collier et al., 2003; World Bank, 2011). This literature often views protracted crises through the lens of humanitarian assistance or economic development policies (Tak et al., forthcoming). However, resolving protracted crises requires solutions that differ from responses to short‐term crisis and non‐crisis development contexts (CFS, 2015). In recent years, Marxist critical scholarship on agrarian studies in war and conflict states has centred around how violence is used as a strategy for resource extraction and labour exploitation in protracted crises. Cramer and Richards (2011, p. 278) contend that mainstream literature has also neglected the agrarian roots and dynamics of violent conflicts, that is, those that are deeply rooted and shaped by ‘agrarian structures, relations and change’. Cramer (2006) and Gómez, Sánchez‐Ayala, and Vargas (2015) argue that the transition to capitalism is a violent one, and the key mechanism in this violent transition is ‘primitive accumulation’, a twin process of forceful asset accumulation and dispossession. Others, such as Thomson (2011), assert that capitalist development can itself be violent and even produce poverty. He suggests that Colombia's protracted conflict has explicit ties with the nation's transition to capitalism and accompanying agrarian questions of how capitalistic forces interact with and reconfigure rural social relations of production. Violence not only impacts the food system but is also created and amplified from within it, making people poorer and more vulnerable and marginalised (Fakhri, 2022). In addition, the environmental security scholarship has been criticised for ignoring the role of political marginalisation in generating violence. Peluso and Watts (2001) centre the role of political economy of access to, and control over, resources in violence generation within the environment (and thus by extension the food system), which in turn is shaped by capital accumulation.

Although the above literature provides insights into the nature of agrarian change, it has not paid adequate attention to the structural causes of crises and the specific ways in which states intervene in the food system. In particular, there exists limited analysis of regions in protracted crises with imperialism and settler‐colonial contexts (Kadri, 2014; Ajl, 2021). When studies pay attention to such contexts, they mostly ignore the ways in which settler‐colonialism and capitalist development interact to produce forms of domination and exploitation (see, for example, Brück, d'Errico, and Pietrelli, 2019; Romano et al., 2020). More recently, Ajl (2021) and Basha (2023) have emphasised the importance of the role of war and imperialism in food systems in protracted crises. Central to this scholarship is the use of the concept of ‘de‐development’ to theorise the specific process of development in occupied or war spaces, such as Palestine, Kurdish provinces, Yemen, and Kashmir (Roy, 1987, 1999; Yadirgi, 2017; and Basha, 2023). De‐development is defined as ‘a process which undermines or weakens the ability of an economy to grow and expand by preventing it from accessing and utilizing critical inputs needed to promote internal growth beyond a specific structural level’ (Roy, 1987, p. 56). The primary objective of de‐development is to disallow independent indigenous development through the dispossession of resources to sustain settler‐colonialism. In this process, the conditions for creating capital accumulation and the extraction of surplus value are stalled, as resources and the means of production are demolished rather than expropriated (Carapico, 2018). Several scholars have highlighted the key mechanisms of agricultural de‐development. For instance, Amira (2021), in his work on Palestine, uses the concept of slow violence to show how the Israeli settler state uses wild boars and sewage waste to curtail agriculture, whereas Tartir (2018) highlights how Israeli policies of access and mobility restrictions through separation walls, checkpoints, and militarisation of agricultural land are used to devastate Palestinian agriculture.

While no agreed definition of protracted crises exists, the Committee on World Food Security acknowledges that the underlying causes of protracted crises include a mixture of conflict, occupation, terrorism, environmental and climate change, inequalities, poverty, and governance factors (CFS, 2015). We argue that existing frameworks inadequately capture changes to indigenous food systems in protracted crises, as there is poor conceptualisation of them. Regions in protracted crises, especially those rooted within colonial and settler‐colonial histories and realities, are often built on the subjugation of marginalised (indigenous/native) groups and their lands. The prolonged and multifaceted impact of recurring violence, environmental change, and socioeconomic fragility manifests both as short‐term shocks and/or longer‐term stressors (CFS, 2015; Tak et al., forthcoming). Tak et al. (forthcoming) contend that while methods to assess short‐term shocks exist, little is known about how to study long‐term stressors. Thus, a fresh conceptualisation of protracted crises that captures the intersecting impact of recurring violence, environmental change, and socioeconomic fragility is required.

In their paper, Tak et al. (forthcoming) take a political economy approach to foods systems in protracted crises and pay attention to structural factors such as settler‐colonialism and the key mechanisms through which settler‐colonialism governs food systems. In particular, the authors stress that land dispossessions and agrarian de‐development are two key mechanisms that create food systems change. Land dispossession is operationalised through land grab and control that decreases access to common and private land, which shifts agricultural production practices away from food to export‐oriented crops. Simultaneously, agrarian de‐development takes place through intensification of export‐oriented crops, de‐peasantisation, disruption to value chains and markets, and closure of traditional market routes, increasing dependency on the settler state. These mechanisms result in the following changes to food systems: (i) a decline in locally‐produced food; (ii) the degradation of the ecology; and (iii) an increase in livelihood vulnerability. Together they delocalise diets and augment market dependency, thereby affecting the nutrition outcomes of indigenous populations (Tak et al., forthcoming). In this paper, we build on the above‐mentioned literature and framework and supplement it with decolonial and interdisciplinary research approaches (see Swiderska et al., 2022) to investigate the impact of protracted crises on Kashmir's food system.

## 3 CONTEXT

Jammu and Kashmir (J&K) consists of three regions, Jammu, Kashmir, and the sparsely‐populated Ladakh, and has been an internationally‐disputed region since 1947 (Korbel, 1967; Bose, 2009; Kanjwal, 2023). Since its provisional accession to India in 1947, the region has gone through distinctive phases of colonisation, military occupation, and settler‐colonialism (Duschinski et al., 2018; Kanjwal, 2023; Zargar and Osuri, 2023). The period between 1989 and 2002 is often regarded as a watershed in Kashmir's political history. After New Delhi rigged the 1987 Legislative Assembly elections, the region turned to armed conflict. A popular insurrection erupted against Indian rule that was brutally suppressed (Bose, 2009; Duschinski, 2009). The period witnessed the initiation of military occupation, with Indian troops taking over civilian spaces, such as public infrastructure, land, and buildings including cinemas and sports complexes (Malik, 2011). North Kashmir Valley was heavily militarised owing to its proximity to the Line of Control^3^ (IPTK and APDP, 2015). From 2002–08, Kashmir Valley was relegated to low‐intensity armed conflict, despite violence continuing and resulting in heavy civilian and militant casualties. In the years between 2008 and 2019, the region witnessed civilian uprisings against the Indian state, characterised by mass public rallies and civilian strikes. During this phase, south Kashmir Valley became a hotbed of resistance (Naqash and Chakravarty, 2016). As of 2015, 700,000 armed personnel were based in J&K, translating to one per 17 people (IPTK and APDP, 2015).

On 5 August 2019, the Indian state re‐annexed J&K by repealing Article 370 of the Constitution of India, which granted semi‐autonomous status to the region. The removal of its autonomy allows non‐locals and outside private companies to acquire large‐scale tracts of land, which was previously not possible owing to Article 370 (Zia, 2020). Scholars have argued that this phase can be characterised as transformation of J&K into a mass surveillance state, deepening of military occupation, and initiation of a settler‐colonial project aimed at changing the demography (Mushtaq and Amin, 2021) and landownership patterns (Zargar and Osuri, 2023). While the colonial/settler‐colonial project in J&K can be traced back to the British presence in South Asia, this paper locates the food system changes in Kashmir in the time since the late 1980s when the region saw heightened militarisation and the eruption of a popular insurgency.

## 4 RESEARCH DESIGN

In this paper, we focus on four rural districts in Kashmir Valley. Bandipora and Baramulla districts in north Kashmir and Kulgam and Shopian districts in south Kashmir were purposefully selected because of the differences in altitude, topography, agro‐ecological conditions, cropping patterns, and distinct political roles in the longevity of the occupation. In the 1990s, the northern districts of Bandipora and Baramulla were the epicentre of popular armed uprisings. In Baramulla district, apple farming was promoted by the state from the late 1950s and was vigorously expanded in the 1990s (Sharma and Raouf, 2016). Bandipora district, meanwhile, is predominantly inhabited by semi‐pastoralists, fisher people, and smallholder farmers, who are more subsistence‐oriented and rely on family labour. In recent decades, the resistance has shifted to the southern districts of Kulgam and Shopian, which have experienced a rise in apple production, especially in the past decade, primarily for household income diversification (Naqash, Wani and Bhat, 2018).

Qualitative interviews were conducted with rural households between July and November 2022. We held 32 semi‐structured and life‐story interviews with a purposeful sample of households. This was supplemented with 13 key stakeholder interviews, including with government officials, agriculture and horticulture scientists, and trade union leaders who represented the interests of the smallholders involved in the horticultural industry and who oversaw the export of its products to Indian markets. The household interviews aimed to capture the linkages between households' food production, commercialisation, market pathways, and dietary practices. The interview schedule also included questions on climate change, conflict, and resilience.

Heightened surveillance of the everyday lives of Kashmiri people since the re‐annexation of 2019 presented many challenges specific to surveillance states, including mistrust and suspicion within the community, disallowing researchers to spend long periods of time at one place. For example, a co‐author of this study had to cut short his doctoral research due to state surveillance.^4^ Studies have discussed the limitations of conducting long‐term ethnography under surveillance and suggest alternative methodologies such as assembling media stories and telephone and Skype interviews (Beban and Schoenberger, 2019; Ryan and Tynen, 2020). Consequently, we relied primarily on our network to conduct interviews in the rural areas, selecting households through a snowball approach. While a relatively small number (32) of core interviews presents limitations, we reached data saturation (Fusch and Ness, 2015; Stratford and Bradshaw, 2021); this was complemented by alternative forms of data, including interviews with actors involved in the agriculture trade and marketing and transect walks (elaborated below), to observe infrastructure development, which captured land dispossession and agrarian de‐development. The analysis was supplemented further by a review of news reports, memoranda of unions, policy documents, and data on government schemes to understand the nature of state policy and the shifts taking place.

In certain places we were cautioned by local interlocuters that conducting interviews would bring us under state surveillance. In such places, we undertook transect walks, which is a participatory method to gather spatial data on an area by observing the surroundings while moving around it (Mahiri, 1998; Mason et al., 2023). For instance, we did a transect walk around military infrastructure and government food storage warehouses with a local respondent. Afterwards, we drew maps with the interlocuter to depict the changes and their impacts on the food system.

## 5 CHARACTERISING KASHMIR'S FOOD SYSTEM

### 5.1 Historicising land access in Kashmir and its role in cultivating a localised food system

Critical to the local food system is the history of progressive land reforms^5^ enacted by the various autonomous and semi‐autonomous state governments since 1947. Not long after the British left the Indian subcontinent in 1947, the then Prime Minister of J&K, Sheikh Abdullah, introduced progressive land redistribution policies. The J&K Big Landed Estates Abolition Act of 1950 was a pivotal legislative measure aimed at rectifying socioeconomic disparities by dismantling large, landed estates and redistributing land among marginal farmers (Aslam, 1977; Prasad, 2014). This legislation imposed a ceiling on landownership of 22.75 acres (besides orchards and grass farms); beyond this, excess land was seized by the government without compensation and redistributed among tenant farmers (Prasad, 2014). Termed the ‘Land to Tiller’ Act, it heralded a transformative phase in the region, emancipating impoverished peasants from feudal oppression while diminishing the influence of landlords and ruling elites (Prasad, 2014; Hamdani, 2016). A significant proportion of marginal farmers and landless labourers acquired landownership rights, and more than 231,000 acres of land was transferred with ownership rights to cultivating peasants free of any encumbrances (Aslam, 1977).

Subsequent to the enactment of the 1950 Act, a series of significant land reforms were introduced in J&K, including the J&K Agrarian Reforms Act of 1976. This legislation, built upon the principles established by its predecessor, aimed to reshape further landownership patterns and tenancy rights within the state, reducing the ceiling on landownership to 12.5 ‘standard’ acres, excluding orchards (Aslam, 1977; Prasad, 2014). Additionally, the Alienation of Land Act of 1938 (Government of Jammu and Kashmir, 1938), predating the Big Landed Estates Abolition Act and resulting from the peasant movement of the 1930s, played a crucial role in regulating the transfer of agricultural land, safeguarding the interests of farmers by prohibiting land transfers to non‐agriculturists and non‐local J&K populations (Ahmad, 2020). The J&K Land Grants Act of 1960 provided a legislative framework for the allocation and leasing of government‐owned land for various purposes, including agricultural, residential, and commercial uses (Government of Jammu and Kashmir, 1960). It empowered the J&K government to lease land for various reasons, including agricultural and allied activities and infrastructure for hydroelectricity, tourism, and other industries (Iqbal, 2020).

The J&K Land Revenue Act of 1996 (1939 AD^6^; Samvat year), enforced restrictions on the sale of agricultural land to non‐locals. Furthermore, it outlined provisions for the eviction of ‘encroachers’, referring to the local people who may take over common land designated for grazing or other public purposes, or where cultivation was prohibited. In such cases, if any structures were erected, the ‘encroacher’ would be removed, leaving a minimum area of 10 *marlas* (approximately 25 square metres) surrounding the structure intact. Effectively, this provision enabled the authorities to reclaim public land while safeguarding existing structures. In addition, if an alleged occupier received a government notice, they had two options: (i) dismantle the structure on the land in question; or (ii) provide an equivalent area from their private property or land acquired for this purpose within the same village (Zargar, 2023).

Likewise, the J&K Common Lands (Regulation) Act of 1956 formalised public entitlements to communal lands while granting local landowners authority over the utilisation of village commons (Khan, 2020). The traditional village communal system evolved into a customary family structure, where landownership rested with families while portions were retained for communal use, known as village common land or *shamilat deh*. The latter encompasses grazing areas where landholders have proprietary rights proportional to their landholdings, allowing them a share based on their property size (Mir, 2023). All of the key land legislations, including those described above, were repelled in 2020 after the re‐annexation of 2019 (Khan, 2020).

Owing to the progressive land redistribution policies that determine the use of commons, most rural households in the region have access to land for kitchen gardens and for grazing animals. In a study conducted in urban Srinagar, researchers found that commons were critical to food security and sovereignty as farmers often relied on both *shamilat deh* and *khalsa* ^7^ to cultivate *haakh*, indigenous collard greens that are a staple of household diets in the Valley (Raja et al., 2023). They also found that the city‐wide geographical spread of *haakh* farms supported urban dwellers during times of curfew, as households could travel short distances to the local farmer's/distributor's house to buy *haakh* instead of going to the markets, which were closed (Raja et al., 2023). In the aftermath of the re‐annexation of 2019, the *haakh* farmers found themselves vulnerable to confiscation of their commons land (Raja et al., 2023) and by extension, their livelihood. The impact of the changes to commons access not only disrupts livelihoods but also has repercussions for food insecurity if food production on commons is restricted.

Kitchen gardens are used to grow vegetables such as shallots, green beans, leafy greens, aubergines, and *haakh*. Often gardens will also contain a few fruit trees, such as peach, apricot, and apple. Kitchen gardens are critical, therefore, to food security in Kashmir, especially so during the long periods of civil curfew and lockdown impressed by the army/state that are a regular occurrence in the region, particularly in the Kashmir Valley. Food is still grown using traditional methods, enabling households to harvest seeds yearly, thus not relying on the market economy for food or for next year's yield. For most of the year, households are not dependent on outside markets for vegetables as they are able to sustain production from their own kitchen garden. Owing to harsh winters in this Himalayan region, households cultivate vegetables in the summer, which are sun‐dried and stored for consumption in the winter. Dried aubergines and bottle gourds are winter staples often cooked with meat. Traditionally, reliance on the market economy for vegetables is limited to harsh winter months, as well as for rice and animal‐sourced foods.

### 5.2 Impact of militarisation on the food system

Since the start of the armed uprising in the region in the late 1980s, the Indian Armed Forces have occupied thousands of acres of agricultural land, forest, and other natural resources. In the decades of the 1990s and 2000s, the state used the military to dispossess peasants of their land on the pretext of national security and counterinsurgency operations and claim territorial sovereignty by military occupation. Despite the decline of armed conflict in the early 2000s, people living in rural areas have experienced militarisation for more than 30 years and have been denied rights to land (IPTK and APDP, 2015; Parvaiz, 2017).

Restriction of civilian movement through the imposition of curfews and checkpoints is a key mechanism through which the occupation disrupts both the functioning of the food distribution network and people's access to food markets for long periods of time (Raja et al., 2023). Often the lockdowns are longstanding, spanning weeks and months. During such periods, the transportation of food products from farms to market is interrupted and workers in the food industry face difficulty commuting. The frequency of market disruption makes the price of and demand for foods volatile (Raja et al., 2023). The military limits civilian movement, often using excessive force to restrict people's ability to travel to markets to purchase food (Hussain, 2022).

Respondents mentioned that farmers and aggregators repeatedly navigate roadblocks by altering routes and changing travel times to circumvent these hindrances. And during the communications blockade of 2019, farmers were also unable to communicate with aggregators to coordinate the collection and delivery of produce. Such circumstances create economic uncertainty, and farmers often lose harvest owing to a lack of cold storage and distribution infrastructure.

The blockades and lockdowns have negatively affected the nutritional security of the population. To assess this, we reviewed district‐wise nutritional data for 2015–16 and 2019–20. In 2016, Kashmir Valley was put under lockdown due to the assassination of Burhan Wani, a popular militant commander—a state lockdown was imposed from 5 July to 31 August (Junaid, 2020). The re‐annexation occurred on 5 August 2019. Even though 2016 was a lockdown year, the restrictions in 2019 were stricter and longer than those imposed in 2016. The re‐annexation lockdown of 2019 continued from August 2019 until March 2020 (ACAPS, 2020). As such, we can use 2016 data as a crude counterfactual to data for 2019 when a specific event took place: the re‐annexation. We compared anthropometric and adequate dietary intake variables for children under five in each district over the two survey years to evaluate the impact of re‐annexation on nutritional outcomes in the region. Relying primarily on short‐term indicators of nutrition, we found that underweight children under five increased across all four study districts from 2015–16 and 2019–20, suggesting a negative impact of re‐annexation in 2019.^8^

The post re‐annexation legislative changes are arguably aimed at dismantling the progressive land reforms to prevent the population from accessing common (agricultural and grazing) lands (Khan, 2020). In 2020, the J&K Development Act of 1970 was amended to facilitate the removal of local government and civilian objections to land allocations to security forces (Javeed, 2020). According to the new amendment, the military can declare any area as strategic. The land under the control of the Indian Armed Forces has been mostly used to accommodate the growing presence of security forces while undermining the local population's access to farmland, pastures, and forest (Bhan, 2020). The policies have also specifically targeted the indigenous transhumanist tribes of Bakkerwals, who have limited access to property rights. This makes it difficult for them to access commons such as forests, pastureland, and meadows for the sheep and goats that they rear. Ironically, the Forest Rights Act enacted in India in 2006, which recognises the right of forest dwellers to public forest resources (which these communities rely on heavily for both livelihoods and habitation), is yet to be implemented properly in J&K (Ganai, 2023). Instead, the state administration continues to expel Bakkerwals from the land they have accessed for centuries. Restricting access to commons has far‐reaching food system implications beyond what is cultivated in kitchen gardens and foraging for wild foods. Rural populations, more so than their urban counterparts, rely on wild harvested plants such as dandelion leaves, mint, mushrooms, and cockscombs.

## 6 SLOW VIOLENCE AGAINST THE FOOD SYSTEM

### 6.1 Displacement of traditional food culture

Over the past three decades, the militarisation of the region has resulted in many legal and illegal land grabs by the military. Official estimates as of 2018 suggest that more than 21,400 hectares of land are under unauthorised occupation of the military apparatus in J&K (PTI, 2018). In addition, the allocation to the army has increased dramatically since the re‐annexation of 2019. One of the easiest targets of the military has been forest land. Various military infrastructure has replaced the lush pine forests. It is alleged that in the 1990s, when the first phase of militarisation began in the region, vast tracts of woodland were deforested so that resistance fighters could not go unnoticed while hiding from the military (Rafeeq, 2020). This capture of forest areas has had a detrimental effect on the local food system.

Respondents from the northern district of Bandipora stated that they relied on the forest to collect the fodder for dairy cows and firewood to cook. The households also foraged for herbs and wild nutritious vegetables with high medicinal properties, including *wopal haakh* (Himalayan Taesal), *what‐kram* (Silene Vulgaris), *gule* (Plantago Lanceolata), and *hand posh* (Circhorium). Forest birds such as the Chukar Partridge (*Kakaw* in Kashmiri) and Himalayan Monal were also part of the diet. However, owing to the presence of the military, locals are fearful of accessing the forests in the same way as they did before. The respondents cited this as a major reason for limiting their access to the forests.

We observed several instances of militarisation of natural resources. We illustrate this using the case of Bandipora district. Surrounding Wular Lake, one of our study sites in Bandipora district is predominantly inhabited by semi‐pastoralists and people relying on the lake for fish, who have historically been dependent on food from natural resources such as forests, water bodies, and pastoral rangelands. Local inhabitants depend on areas surrounding the lake for grazing cattle and harvesting the regionally famous water chestnuts, all critical for earning a living. Households interviewed in the area owned little land for the primary purpose of housing. Owing to the nature of the lake area, these households had received rights to cultivate the wetlands surrounding the lake. They principally cultivated vegetables, paddy, and maize on this land. In 1949, under the Grow More Food Scheme, marshland, locally known as *rakhs* land (cultivable wastelands), was allotted to landless peasants with partial rights. In return for access to the *rakhs* land, the peasants had to give one‐quarter of the produce to the Department of Rakhs and Farms (High Court of Jammu and Kashmir and Ladakh at Srinagar, 2023).

**FIGURE 1:**
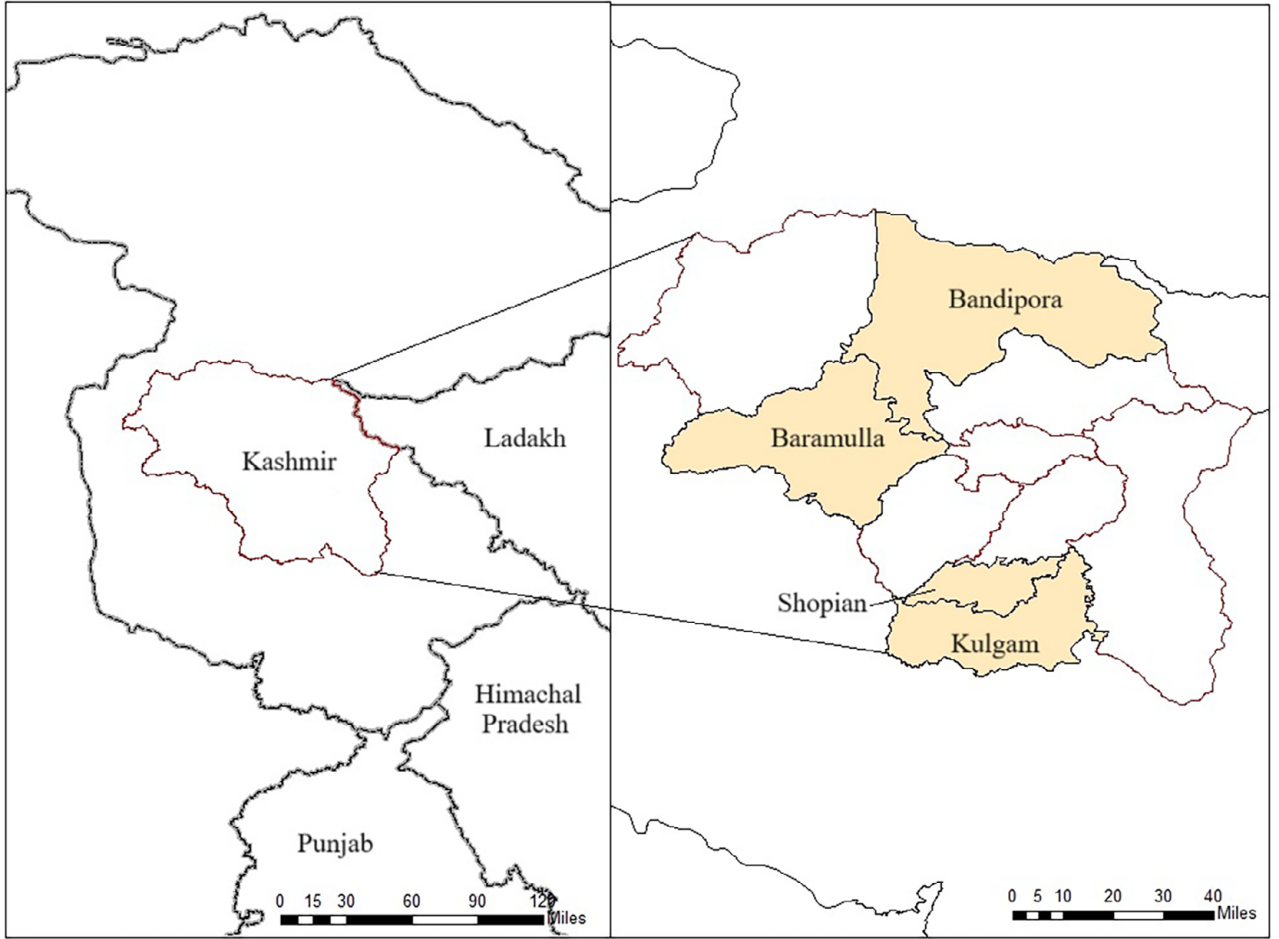
Map of Kashmir to show the field sites **Source:** authors.

**FIGURE 2:**
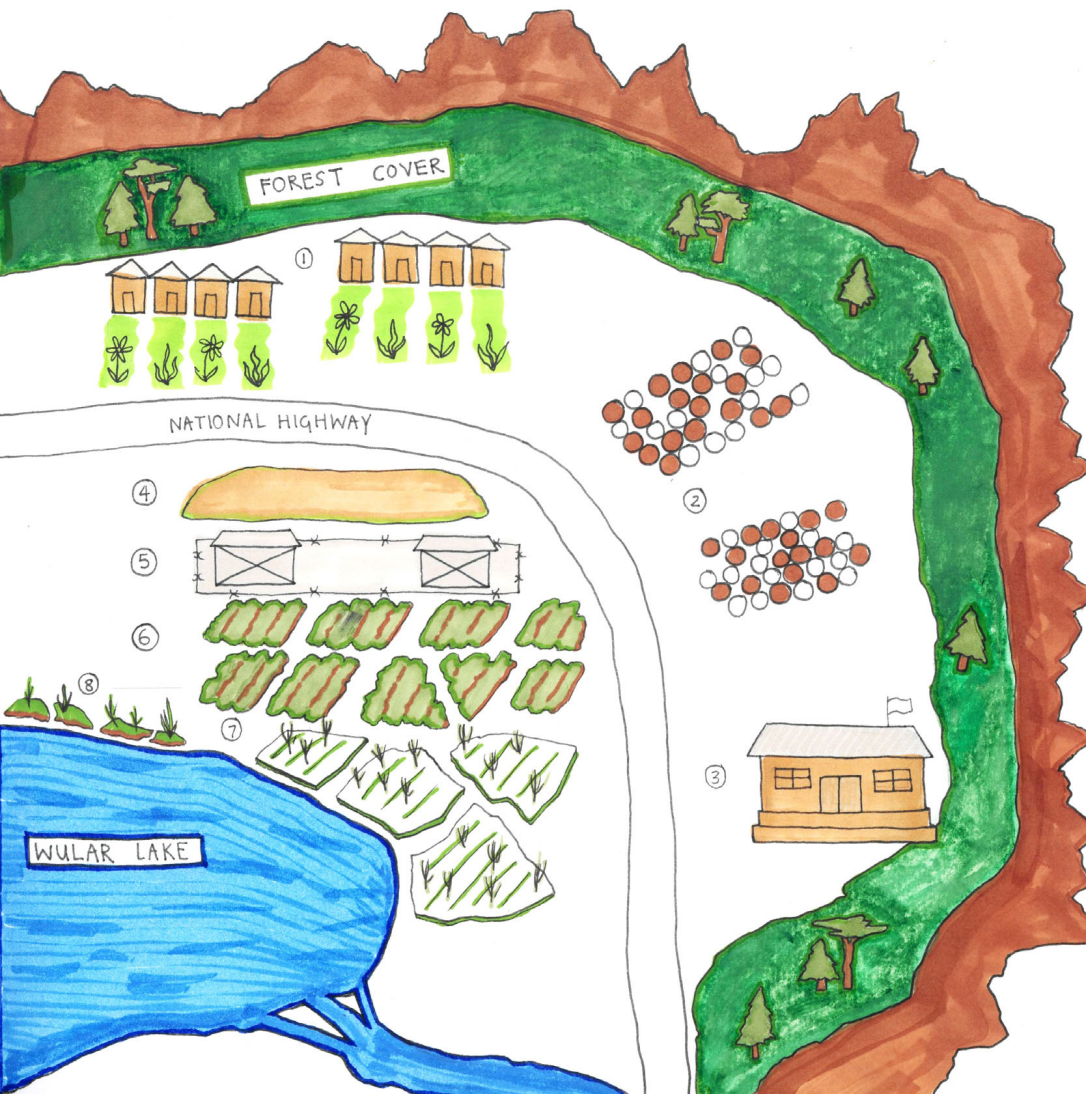
Drawing showing the relationship between structural violence and changes in the food system. **Notes**: point 1 is the houses of the villagers and kitchen gardens; point 2 is the apple and walnut orchards; point 3 is the Food Corporation of India warehouse; point 4 is common grazing lands; point 5 is the military cantonment; point 6 is the low‐lying vegetable cultivation lands; point 7 is the low‐lying paddy cultivation lands; and point 8 is the *rakhs* lands. **Source:** authors, field notes.

Figure 1, 2, 3 maps the impact of increased militarisation and infrastructural development on the food system, captured through transect walks in Bandipora district. While traversing the area, we saw large permanent structures on previously commons land. New buildings included military infrastructure, eco‐tourism projects around Wular Lake, and an existing state‐run Public Distribution System (PDS) warehouse which stores food procured from outside the region. We also documented the area where military infrastructure has been built, its connections to farmlands, and how this has disrupted the agrarian culture of the villages. The figure maps the village houses and their low‐lying paddy farms, along with land used for horticultural purposes, such as for walnut and apples trees and kitchen gardens. There also exists a Food Corporation of India (FCI) storage facility. Permanent military infrastructure surrounded the banks of Wular Lake to accommodate the growing presence of security forces. This has reduced grazing land for locals, affecting livestock and dairy production in the area. In particular, the respondents mentioned that until the 1990s, when militarisation intensified in Kashmir, their kitchen gardens, primarily worked on by women, were next to their orchards and paddy land. With the increase in the military presence, the kitchen gardens were shifted from agricultural land to within the household compound. The displacement of common land has had a gendered impact on women's participation in agricultural activities, which depend heavily on feminised labour. Respondents declared fear among female members about accessing their farmland and other natural resources when military infrastructure was in close proximity to the resource. One said:

*In the past, the households of our village were dependent on forests for food, wood, and coal. Most of the young women used to leave early in the morning to go to the forest. Besides keeping me physically fit, I found spiritual and psychological comfort in going to the forests. We also used to collect wild foods [vegetables and medicinal herbs] from the forest. The tradition of women going to forests started diminishing in the 1990s as forests became militarised, and now younger generations are not interested in going to forests. . . . Earlier, we had a vegetable garden far from our residential house, but in recent decades, they have shifted it, just adjacent to our house. They shifted it due to the army, as some incidents happened in the past, and they now don't have a vegetable garden far from their house*.

At the time of the fieldwork in 2022, a new military cantonment (see point 5 in Figure 2) was being built on common land where people grazed animals and cultivated vegetables and paddy. The taking over of village common land has disrupted the use of commons as a space to produce food (see point 4 in Figure 2). Once functioning, the location of the new military cantonment is such that it will dominate the surrounding low‐lying private land used by the villagers (especially women) for vegetable and paddy farming (see points 6 and 7 in Figure 2).

**FIGURE 3:**
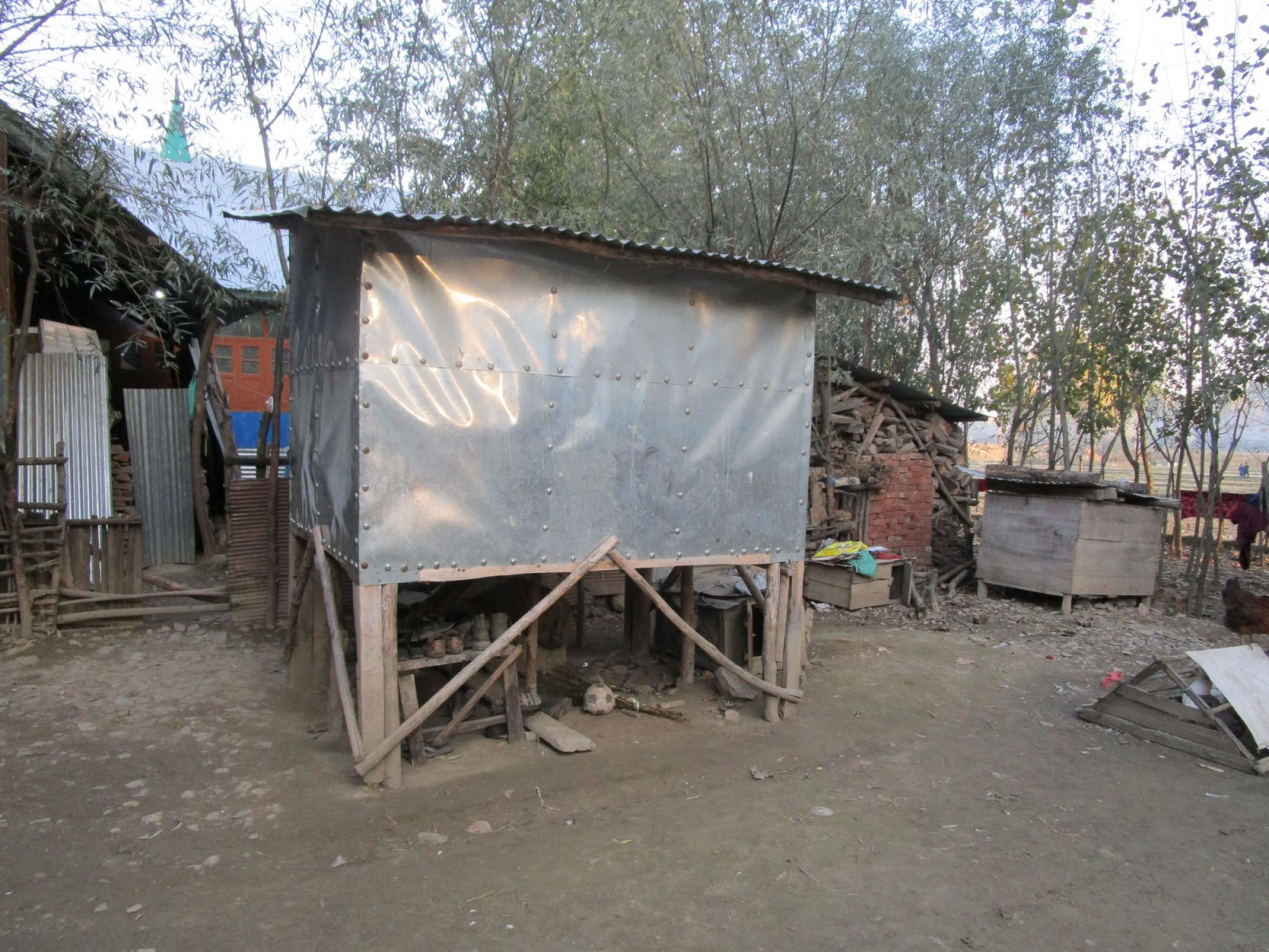
Grain room (*daan kuch*) at a paddy farmer's home compound **Source:** authors.

With the ever‐increasing military encroachment and declining access to farmland and commons, dependency on Indian government‐run non‐indigenous rice distributed via the PDS and on private markets has risen. Figure 3 shows a *daan kuch*, a traditional grain room in which to store rice, at a paddy farmer's home compound. We found these to be slowly disappearing and FCI warehouses and shops to be taking over the food system landscape.

Respondents narrated incidences from the 1990s when the Kashmiri armed resistance was at its peak. Military troops disguised as *bhoot* (ghosts) attacked farmers working in agriculture fields, creating fear among people and deterring them from visiting their farms.

### 6.2 Increased reliance on markets and economic uncertainty

A shift from food (paddy and mustard) to commercial agriculture (particularly apples) has increased reliance on food imports from India and household reliance on market pathways. Smallholder farms are gradually disappearing or are being incorporated as part of commercial agriculture. For instance, Kulgam district was titled the Rice Bowl of Kashmir, given its high production of indigenous rice varieties, but has seen a significant decline in rice cultivation: between 2002 and 2021, 50 per cent of the paddy land has been converted to horticulture, from 44,000 to only 22,000 hectares (Gul, 2021). A trend towards decreased prioritisation of food production for local consumption was observed in Baramulla, Shopian, and Kulgam districts. A consistent theme across the household interviews in the southern districts was that farmers are moving from traditional paddy and horticulture cultivation to high‐density apple orchards. This shift has led to increased food dependency on government‐run ration shops and private markets that procure supplies from outside of J&K. One respondent remarked:

*Ten years ago, we transformed our paddy land into an apple orchard. As it's impossible here to keep paddy fields in the middle of apple orchards, we decided to cultivate apples. Earlier, we used to get everything from our land. The waste from food was used as fodder for animals. Now we get everything from the market*.

Farmers were uprooting old varieties of traditional apples and replacing them with high‐density varieties to commercialise horticulture further. The increased implementation of neoliberal policies in the region—characterised by deregulation of the use of agricultural land for commercial purposes, a push towards production of cash crops, the absence of minimum support price for key agricultural crops like apples, the deregulation of low‐cost agricultural imports, subsidies for corporate investors, the facilitation of contract farming, and market‐led private control of seed, fertiliser, and pesticide procurement—is primarily driven by the Indian state's economic and political interests and as a means of economic subjugation. The intensification of agriculture through neoliberal policies involves the dispossession of indigenous people's land and the loss of seed sovereignty in favour of national and foreign companies and agro‐industrialists (Sansour and Tartir, 2014). Farmers are often pushed towards export‐oriented agriculture, requiring the development of new marketing and trade infrastructure to connect with globalised markets.

The most important paradox specific to protracted crises is how settler‐colonialism restricts agricultural production and marketing. Often, in the contexts of protracted crises, the state resorts to supply chain disruptions through the closure of critical infrastructure such as highways, ports, entryways, borders, and existing trade routes. In the case of Kashmir Valley, the Indian state frequently resorts to economic blockade through the closure of highways to curtail the food supply and marketing of exported goods. Since 2019, the Indian government has halted the movement of trucks transporting apples on the only highway that connects Kashmir to India. These barriers to outside markets have persisted and have forced the smallholder apple farmers to sell their produce below the market rate. The value chain has also been adversely affected by India's trade policy of dumping cheaper Iranian apples on the market (Maqbool, 2022).

## 7 DELOCALISATION OF DIETS

Traditional livelihoods are rapidly being destroyed, leading to forced changes in diets. As a result of increased reliance on non‐local food that is high in carbohydrates and fats, people are unhealthy and nutritionally vulnerable. One respondent stated:

*Before, we used to self‐do all the work in our agriculture fields. Exercise used to happen while working in fields. Now most people are facing high blood pressure, diabetes, and obesity, and these are the direct results of inactivity*.

While the changes to Kashmiri diets may not be a deliberate policy of the occupation, they are non‐direct consequences of the protracted crises. For indigenous peoples that rely on natural resources such as forest and land, non‐direct change shifts land use patterns: land is repurposed for the cultivation of cash crops or for other economic activities, which means it is no longer available for one's own food production (Kuhnlein and Receveur, 1996). This leads to delocalisation of food supply. Delocalisation is defined as the ‘processes by which food species and varieties, production techniques, and use patterns disseminate throughout the globe’ (Kuhnlein and Receveur, 1996, p. 423).

The delocalisation of food supply is a critical way in which the Indian state has manufactured Kashmiri dependency on the country. This delocalisation stemming from dependency on PDS rice and the shift away from food production to horticulture are major determinants of dietary change: an ever‐increasing proportion of a typical meal is coming from distant sources that rely on markets. This in turn also has implications for the food culture, moving from reliance on local produce to more processed and far‐travelled foods. A similar pattern to that in J&K has been documented in the neighbouring region of Ladakh, a highly militarised and strategic border zone, where there has been a drastic shift from self‐sufficient food grain production to increased food imports procured by the PDS from the plains of India (Pelliciardi, 2013; Bhan, 2014).

As compared to previously when Kashmiris would have produced, grown, or cultivated their own food, the change in the use of land from kitchen gardens and paddy cultivation to orchards full of non‐local varieties of apples, kiwifruits, and grapes means that overall, people have less food to eat unless it is bought from Indian markets. Under normal circumstances, where increased marketisation of food, and thus improved agricultural productivity, may lead to economic growth and subsequently development^9^ (Ahluwalia, 1985; Prowse and Chimhowu, 2007; Lanjouw and Murgai, 2008), in settler‐colonial contexts, reliance on markets makes the population vulnerable to disruptions, limiting access to food, as markets are controlled by the state. The state's promotion of the commercialisation and marketisation of agriculture has manufactured dependence of imports and exports on settler market. This market dependency is used as a tool of control and domination. In Kashmir, during periods of mass civilian protest, economic blockades by the Indian state that limit the inflow of essential food supplies and the outflow of exported cash crops are used as means to subjugate people, forcing them to surrender and thus breaking the cycles of mass resistance. In non‐settler‐colonial contexts, a shift from subsistence to the commercialisation of agricultural production could be welcomed^10^ and termed as part and parcel of the neoliberalisation agenda. In settler‐colonial contexts, while increased commercialisation may seem to present opportunities to integrate into global value chains for food commodities and hence improve livelihoods, in practice, this integration provides the settler state with access to—and control of—the local economy. Consequently, this gives the state the power to disrupt economic activities to de‐develop the agrarian economy. This is where a classic neoliberal state and a settler‐colonial state depart from each other: for a neoliberal state, the end goal would be to increase activities such as agricultural commercialisation through primitive accumulation, whereas for a settler state, the accumulation of land is not an exercise in primitive accumulation to create the conditions necessary for development (Tak, 2023), but rather a way to dispossess the local population of the means of production in order to advance the settler‐colonial project.

## 8 INHERENT RESILIENCE IN THE FOOD SYSTEM

In the preceding sections, we have shown how protracted crises in Kashmir impact the food system. Building on them, this section looks at how rural households cope under the long‐term uncertainty of protracted crises. We have historicised progressive land reforms in J&K, which were critical in shaping access to land within households through kitchen gardens and commons, meaning that most households are able to grow fruits and vegetables. This practice, which is facing several challenges following re‐annexation, is critical to the resilience of Kashmir's food system, especially as access to food markets can be prohibited at any time, creating disruptions to food value chains. In this study we wanted to understand better the importance of these kitchen gardens and the nexus between one's own production and reliance on markets for food acquisition.

During our fieldwork, we examined how households utilise their kitchen gardens and under which conditions they rely on markets. Of the 32 rural households we interviewed in the four rural districts of Kashmir, 27 had access to a kitchen garden. The remaining five households were fisher people living close to Wular Lake who, as mentioned above, also had access to cultivable *rakhs* land. We documented that *haakh*, potatoes, green chillies, tomatoes, radish, cauliflower, garlic, bottle gourds, brinjal, pulses, cockscomb flower, round guards, and fennel were cultivated. At the household level, women played an important role in farming vegetables in the kitchen gardens as well as preserving seeds and sun‐drying vegetables. Households showcased resilience by using traditional production and storage methods. They were aware that the increasing dependency on markets contributed to the rise in monthly costs and did not welcome this shift.

Cultivation was done under low‐input conditions in an organic and sustainable manner, with seeds harvested every year. Almost every household we interviewed had storage vessels inside the house for stowing large quantities of food, especially rice that would last for months and, in some cases, for a year owing to the frequency of unexpected curfews and lockdowns. The logic behind hoarding food for future needs seems to overcome the uncertainty and fear generated by the protracted crises. Even cash crop‐cultivating households reserved a portion of land for a small kitchen garden within the house compound boundary.

### 8.1 Communities of care

In Kashmir, the indigenous communities, through the promotion of home gardens, preservation of seeds, and sharing of food, employ what Scott (1985) terms as ‘everyday forms of resistance’. These are important to their quest for self‐determination and resistance to ongoing colonisation, dispossession, and erasure. Produce from the kitchen garden was consumed within the household and excess was often shared with neighbours and relatives when food supplies were blocked both as a form of collective punishment and deterrence against resistance by the people. During lockdowns and in the aftermath of the 2014 floods,^11^ food, especially vegetables and rice, was collected in the rural areas and redistributed in the capital city of Srinagar, sometimes overnight. This is an example of communities of care at the macro level that have sustained and prolonged resistance in urban areas where people have limited means to cultivate food. One respondent noted:

*Conflict has had a positive impact on our food system as we have developed a culture of sharing. Here, people have evolved to help each other in dire times rather than dwarfing into self‐centred survival mode. At the time of uprisings, people act as a unit. During [the] 2008 and 2010 uprisings, I was active at the village level. At the village level, it was a snowball process—if one person used to take [the] initiative of collecting food, then five to six people used to get together and collect rice and vegetables*.

Interviewees indicated that in rural Kashmir, there is a culture of solidarity established through sharing food. People collectively fight food insecurity at the time of uprisings. We found that the mosque as an institution played an important role at the village level in combating food insecurity through *Bait‐ul‐Mal*,^12^ a form of collectivisation. Farmers gave a portion of their agriculture produce to the local mosque, which then redistributed food among the poor and underprivileged sections of society. One respondent described this as follows:

*Conflict has given us experience of how to overcome difficult situations such as the one in floods [in 2014]. Instead of hoarding food, people shared food. Community treasures (known as Bait‐ul‐Mal) formed in local mosques use donations gathered through charities to offer food items, household essentials, or cash to needy families. Other people tap local fundraising to help daily wage earners and vendors hit by the economic impacts of the curfews, floods, or successive pandemic shutdowns. We were not shy in going to households and asking for food [collecting food]. My mother used to tell me to take this [bottle] gourd and rice and it should reach Srinagar*.

The study participants also talked about the tactics that the state employs to intimidate and silence the people who organise the community to fight food insecurity collectively at the time of uprisings. As one respondent pointed out:

*As a counter strategy to food collection and redistribution during uprisings, the state security agencies harassed and arrested the key organisers and, in several instances, the collected food was snatched from them. They were harassed and were asked to provide details of the food collected*.

Furthermore, respondents mentioned many instances of where the army destroyed households' stored supplies of rice by mixing it with kerosene and broken glass. The targeting of people involved in community collection and distribution of food, as well as of food supplies, and the disruption of value chains should be seen as deliberate mechanisms to deny food access to communities to break their resistance.

In an environment where hunger is used as a weapon of war, smallholders make significant strides towards restoring their relationships with the land and their food, improving their health, and advancing their political and economic success. The indigenous food systems should therefore be understood not merely as a site of growing marginalisation, but also as one where native populations resist through the production and promotion of local food. The survival strategies exemplify Scott's (1985) ‘everyday forms of resistance’, as smallholder farmers, despite structural constraints, promote local production to fight food insecurity and manufactured dependence on markets.

In this paper, we see resilience as ways to resist changes to food systems owing to militarisation. Our fieldwork suggests that activities that resist food system changes are often coping mechanisms or adaptation strategies based within cultural and indigenous knowledge economies to oppose and disrupt the settler‐colonial project in indirect ways. An example is the shifting of kitchen gardens from agricultural land to within the home compound due to the rising militarisation of land. As such, kitchen gardens play a critical role in supporting food security during curfews and lockdowns. Also notable is the role of communities of care in collecting and distributing food from rural to urban areas, which prolonged the length of popular uprisings, such as the one during summer 2016 that lasted six months, with at least 53 days of government curfew. The act of redistribution of food disrupts the settler‐colonial project by providing vital sustenance during long‐term lockdowns that are often used as collective punishment by the state and military.

## 9 DISCUSSION AND CONCLUSION

In this paper we have highlighted the role of resource grabs as part of militarised settler‐colonialism in driving food system change and increasing vulnerability to food insecurity in protracted crises. Our findings show that the key drivers of food system change in Kashmir's protracted crises are, first, that intensive state‐backed promotion of cash crops has increased reliance on food imports from mainland India and augmented household reliance on market pathways. This is critical because income from cash crops highly fluctuates annually owing to the economic uncertainties created by the settler‐colonialism and militarised shutdowns in the region. Second, militarisation of natural resources and everyday slow violence (Nixon, 2013; Braverman, 2023) dispossess the local populations of access to public resources. These drivers of food system change have decreased the local population's access to commons land to grow their own food, fuelling delocalisation of diets and thus increased dependency on food imports. In areas marred by protracted crises, this dependence on external markets and the market economy has perilous implications owing to the economic uncertainty created by occupation militarisation and/or settler‐colonialism.

Kashmir Valley is not alone in experiencing the destruction of the local food system through militarised land grabs and the neoliberalisation of agriculture approaches. In the neighbouring region of Ladakh, evidence of direct delocalisation of traditional diets due to militarisation has been documented. Dame and Nüsser (2011) and Pelliciardi (2012) have demonstrated that eating habits in Leh district changed because of the cheap, subsided availability of rice and wheat flour imported through the PDS, discouraging farmers from grain production of buckwheat and barley for self‐sufficiency. Bhan (2014) found that the increased employment of indigenous Brogpas people in the Indian Army shifted labour away from the cultivation of barley or buckwheat to horticulture cash crops such as fruits and vegetables, which were sold to the military, thus moving food cultures away from a diet of barley to rice, which is subsided through the PDS. The said studies illustrate that while increased reliance on the army for the sale of fruits and vegetables could earn income, it also boosted reliance on markets for the purchase of staple foods (Pelliciardi, 2012; Bhan, 2014) and changed social structures between local indigenous groups, as customary food and labour‐sharing arrangements between Buddhist and Muslim Brogpas weakened due to the availability of cheap rice from the PDS (Bhan, 2014). As such, not only were diets delocalised but communities of care were destabilised.

The food system changes are packaged as implications of economic development by the settler state to disallow self‐governance. This has been framed as agricultural de‐development in other locations, such as Palestine (Roy, 1987). The militarisation of productive public assets such as agricultural land and other natural resources deepens this de‐development of the region. It constitutes a strategy of dispossession without accumulation, de‐peasantisation, loss of livelihoods, and forced commoditisation, severely affecting the food sovereignty of the peasantry (Nabi and Ye, 2015; Tak, 2023).

The food system changes in Kashmir need to be analysed in the historical context of the region being a frontier zone that is witnessing a popular self‐determination movement. In recent years, new forms of militarisation of natural resources have been an integral part of the Indian state's strategy of creating conditions to expand and deepen its reach in the region. It is challenging for locals to resist land dispossessions owing to the military's coercive role and surveillance. The military apparatus plays an important role in agrarian transformation by destroying local patterns of food production and consumption. The enforced dispossession and marginalisation of rural households strengthen the settler project, which is intrinsically linked to the destruction of the local food system. The economic development approach in Kashmir ignores the structures of power and dominance that are inherent in the process of development, where the power of a ‘military state’ to control the indigenous population through extreme militarisation, uninterrupted long curfews, and mass surveillance plays a major part in countering and blocking alternative paths of development. In fact, the Indian state has utilised the development agenda to override Kashmir's political society and restricted local engagement in decision‐making regarding economic development.^13^

The paper also sheds light on everyday forms of resistance or the inherent resilience of the food systems under protracted crises. We highlight how households in Kashmir, through the preservation of indigenous agrarian knowledge, have taken significant steps towards breaking down the food dependency forced by the state. These knowledge economies are the key to promoting indigenous food systems under protracted crises; however, they are facing multifaceted challenges that threaten the potential to realise the right to one's own food. Access to land and natural resources and the sovereignty to decide on one's own food policies are indispensable for the promotion of indigenous food systems.

More broadly, the paper argues that in settler‐colonial contexts the state and its military apparatus play an important role in agrarian transformation and the creation of protracted crises, by destroying local patterns of food production and consumption. These actors use neoliberal policies as a tool to deepen state control. The paper adds to literature on critical agrarian change (see the works by Roy, 1987, 1999; Kadri, 2014; Ajl, 2021; Basha, 2023), environmental security^14^ (Peluso and Watts, 2001), settler‐colonialism (Wolfe, 2006; Veracini, 2017), and indigenous studies (see, for example, Kēhaulani Kauanui, 2016). In particular, we depart from literature on the agrarian roots of violent conflict that argues that agriculture expansion for capitalist accumulation and exploitation is a central driver. Instead, we assert that settler‐colonialism transcends the neoliberal project of capitalist accumulation, where the state does not engage in primitive accumulation. This is because, under de‐development, the conditions for establishing capital accumulation and the extraction of surplus value are stalled, as the resources and the means of production are demolished rather than expropriated (Carapico, 2018).

To conclude, the paper contends that a state‐led de‐development strategy, especially in the form of slow violence and agrarian transformation, is a key driver of food system change that fuels the delocalisation of Kashmiri diets. The Indian state and its military apparatus play an important part in agrarian transformation by destroying local patterns of food production for consumption. As such, the paper presents a fresh and critical approach to researching food systems in protracted crises.

## CONFLICT OF INTEREST

We declare no conflict of interest.

## ETHICS STATEMENT

This paper reports analysis of primary data. Persons from whom data were collected gave their free, prior and informed consent. The data obtained has been kept confidential and used anonymously.

## FUNDING STATEMENT

We acknowledge the Cabot Institute for the Environment, University of Bristol, for its support through the Innovation Fund, and the Independent Social Research Foundation for its small group project grant.

## Data Availability

Research data are not shared.^15^

## Endnotes

^1^
Raja et al. (2023) use ‘the phrase *protracted crisis* to draw attention to the duration of the political conflict, the power asymmetry between state and civilian actors, the weakened institutions, and adverse health outcomes in the study setting’. While existing literature uses *crisis* in its singular form, we use the plural in this paper to highlight the multifaceted nature of long‐term crisis, including violence, poverty, and environmental change. See the second section for the definition of protracted crises used in this paper.

^2^
See, for example, Alinovi, Hemrich, and Russo (2008), FAO (2012), and CFS (2015).

^3^
The Line of Control is the de facto border between India and Pakistan.

^4^
See Zargar (2022) on threats to Kashmiri scholars conducting research in the region.

^5^
A full history of land reform in J&K is beyond the scope of this paper; however, we present key legal doctrines that have over time determined access to land in the region.

^6^
Under Dogra rule (1846–1947), the Samvat calendar applied, which is 56–57 years ahead of the Gregorian calendar.

^7^
State‐owned public land is known locally as *khalsa*.

^8^
Whilst we have compared Demographic and Health Survey (DHS) data for 2016 and 2019, a Working Paper by Agrawal and Kumar (2017) has raised concerns about the reliability of data for peripheries such as Nagaland and J&K, owing to faulty sampling frames, sample non‐coverage, and biased samples. It is also speculated that collecting DHS data during lockdowns may be difficult. However, the Indian Army regularly conducts household census and surveys in J&K for surveillance (Singh, 2024). Under relevant conditions, therefore, data collection is possible. Even with its potential limitations, the DHS is the best available data on nutrition in J&K. For this paper, we assess the short‐term indicators of nutrition as the survey data relies on a large number of surveyed households. Furthermore, our fieldwork qualitatively backs up the deterioration in nutrition.

^9^
This may be unequal development, however.

^10^
By advocates of economic development policies. Perhaps less so by those who research the social justice impacts of neoliberalisation.

^11^
In summer 2014, the Jhelum River, which laces through Srinagar, overflooded and engulfed a large part of the city. Rescue operations were slow in reaching the affected local population.

^12^

*Bait‐ul‐Mal*, which literally translates as the house for money, is an informal collective public treasury used for social and welfare provisions.

^13^
See, for example, Zia (2020) and Tak (2023).

^14^
We agree with the assertion of Peluso and Watts (2001) that conditions of resource scarcity do not have a monopoly on violence and that the political economy of access to resources has a role to play in how violence shapes the environment.
